# Investigating the Icr Effect in a Zhadin’s Cell

**Published:** 2009-06

**Authors:** L. Giuliani, E. D’Emilia, S. Grimaldi, A. Lisi, N. Bobkova, M. N. Zhadin

**Affiliations:** 1 *ISPESL-Istituto Superiore per la Prevenzione e la Sicurezza del Lavoro, Centro Ricerche di Monteporzio Catone, Roma, Italy;*; 2 *CNR, Istituto di Neurobiologia e Medicina Molecolare, Tor Vergata, Roma, Italy;*; 3 *Institute of Cell Biophysics, Poushchino, Moscow Region, Russia*

**Keywords:** iono-cyclotronic resonance (ICR), BLZ, Zhadin’s cell

## Abstract

Investigations into the ion cyclotronic resonance (ICR) in living matter confront the so called Zhadin effect (12), whose explanation is not fully achieved. Several attempts have been done to explain this phenomenon, the most interesting of which is based on Quantum Electrodynamics (18): the molecules of water, the ions and the biomolecules form extended mesoscopic regions, called Coherence Domains (CD), where they oscillate in unison between two selected levels of their spectra in tune with a self-produced coherent E.M. field having a well defined frequency, dynamically trapped within the CD. Moreover, it is possible, to induce, by an external applied field (either hydrodynamical or EM) or also by a chemical stimulation, coherent excitations of CD’s that give rise to electric currents circulating without friction within the CD’s: as a consequence magnetic fields are produced. A resonating magnetic field thus is able to extract the ions from the orbit and push them in the flowing current. Electrochemical investigation of the system suggested that the observed phenomenon involves the transitory activation of the anode due to ICR, followed by anode passivation due to the adsorption of amino acid and its oxidation products (18). This hypothesis induced us to investigate an alternate configuration of the experiment, removing the electrolytic cell and submitting a flask containing the solution into a condenser to be exposed to the proper ICR. Temperature and variable parameters involved in the effect have been investigated in order to overcome the randomness of the effect.

## BACKGROUND

When we apply orthogonally to a ionic current a combination of two parallel fields, one static, B_o_, the other one alternating, B_a_, at an appropriate frequency between 1 and 100 Hz, whereas the B_a_ is one thousand times lower than the B_o_, an arising ion current peak can be observed. The effect can be detected only if the frequency matches the so called cyclotron frequency of the involved ion species, which is proportional to B_o_ and its duration lasts some seconds after the alternating field is applied. The effect, first investigated by A. Liboff (6), was invocated to explain important experimental results showing non thermal interactions between magnetic fields and living systems (2, 3).

In the enusing ninety, years a new approach to this matter, first due to M. Zhadin, has shown that the increasing ion current effect concerns not only metallic ion species but also organic ions and nucleic acids (10, 12).

Several attempts have been made to explain this phenomenon, the more interesting of which is the explanation provided by E. Del Giudice, G. Preparata, M. Fleyshman and G. Talpo in 2002 (15), in the theoretical frame of Quantum Electro- Dynamics (QED) (9).

According to this explanation: “in living matter the molecules of water, the ions and the biomolecules form extended mesoscopic regions, named Coherence Domains (CD), where they oscillate in unison between two selected levels of their spectrum in tune with a self-produced coherent E.M. field having a well defined frequency, dynamically trapped within the CD. It is possible, moreover, to induce, by an external applied field (either hydrodynamical or EM) or also by a chemical stimulation, coherent excitations of CD’s that give rise to electric currents circulating without friction within the CD’s: as a consequence magnetic fields are produced. A resonating magnetic field is thus is able to extract the ions from the orbit and push them in the flowing current” (15).

Partially reforming this hypothesis, a reduction of the phenomenon to electrochemical issues was suggested: they were investigated “the mechanisms that were at the origin of the so-far poor reproducibility of the above effect: the state of polarization of the electrode turned out to be a key parameter. The electrochemical investigation of the system shows that the observed phenomenon involves the transitory activation of the anode due to ion cyclotron frequency effect, followed again by anode passivation due to the adsorption of amino acid and its oxidation products.” (18).

The above hypothesis induced us to investigate an alternate configuration of the experiment, removing the electrolytic cell and submitting a flask containing the L-glutamic acid solution into a condenser to be exposed to a weak magnetic field matching the L-glutamic acid ion cyclotronic frequency. Furthermore more attention was given to the variable parameters involved in the Zhadin’s experiment in order to overcome the intrinsic randomness of the effect (17, 18).

## MATERIALS AND METHODS

**Figure 1:**
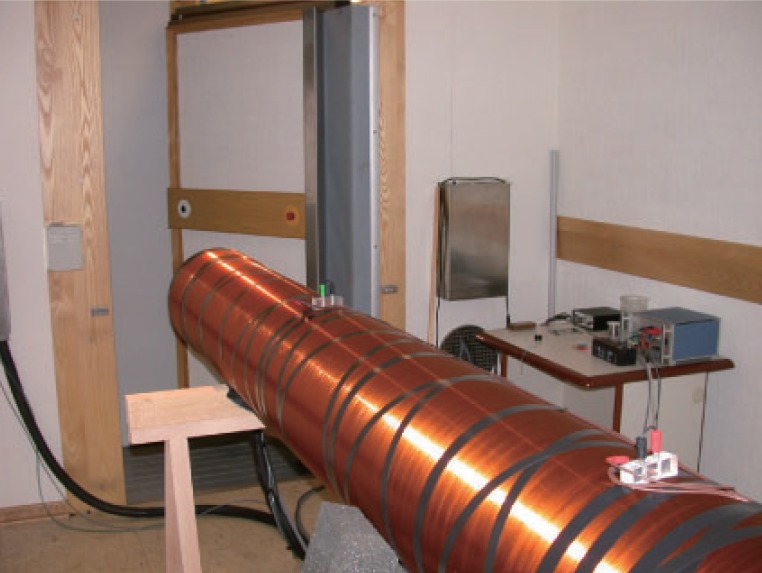
The great solenoid in the shielded room.

A shielded room, for the earth static magnetic field and for industrial electromagnetic noises, has been built at the Tor Vergata Center of National Council for Researches (CNR), close to Rome, where our experiments are performed. A thermostated equipment within a large solenoid (Figure 1) and proper electromagnetic probes have been developed (patent pending due to CNR and ISPESL) to provide both static and alternating magnetic fields suitable to expose cells or Petri disks and to detect the arising Zhadin effect, under controlled temperature, humidity electromagnetic conditions.

The large solenoid (3 meters long and 33 cm da) has been properly powered in order to continuously generate low magnetic field frequencies from 0 to 10 Hz. The alternating magnetic field is combined with a static magnetic field reproducing the earth magnetic field. In such way the parallelism of the two combined magnetic fields is ensured and the intensity of the static field is fixed (48.9 μT), in order to provide the appropriate cyclotronic frequency of the concerned aminoacid.

**Figure 2:**
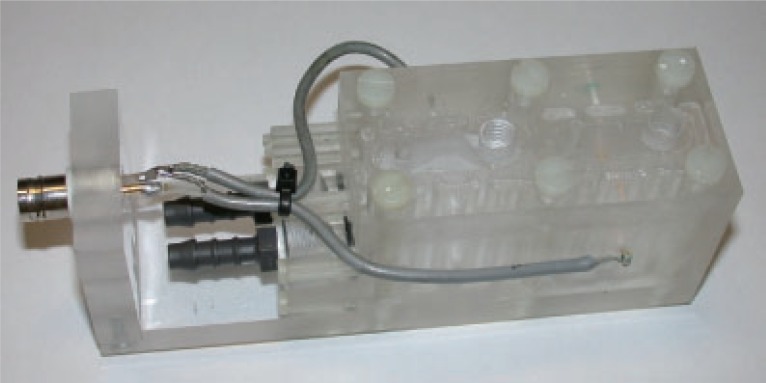
The Zhadin’s cell.

**Figure 3:**
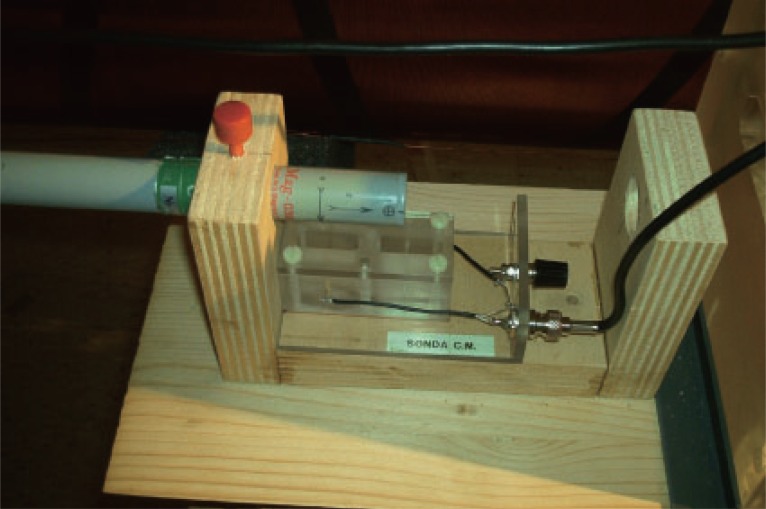
The probe and the slot with the electrolytic cell.

First the *classical* Zhadin experiment has been replicated several times in order to get the full reproducibility: an electrolytic cell with two gold electrodes, containing a glutamic acid solution (33 g/l) at acid pH, has been exposed to the combined static and magnetic fields. A Zhadin’s cell has been employed like the one in Figure 2, placed in the slot under the probe, as in Figure 3.

**Figure 4:**
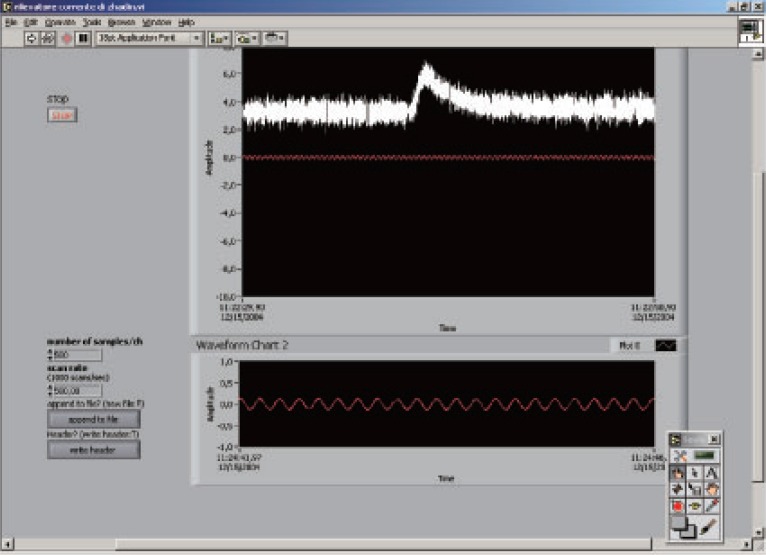
The Zhadin effect detected on Dec.12^th^ 2004.

The full reproducibility, according to practical restrictions, has been reached (Figure 4) even new experiments are to be attempted in order to provide a large data base of the parameters involved in the Zhadin effect.

**Figure 5:**
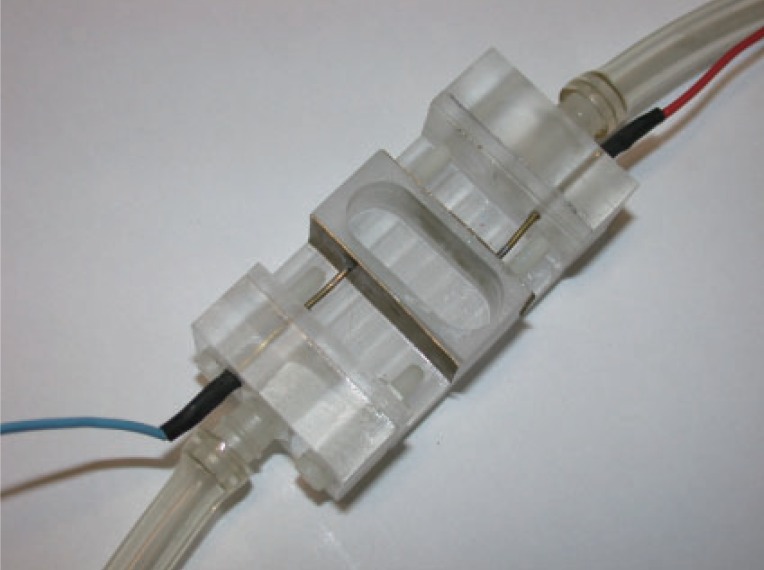
The flask within the external brass condenser.

**Figure 6:**
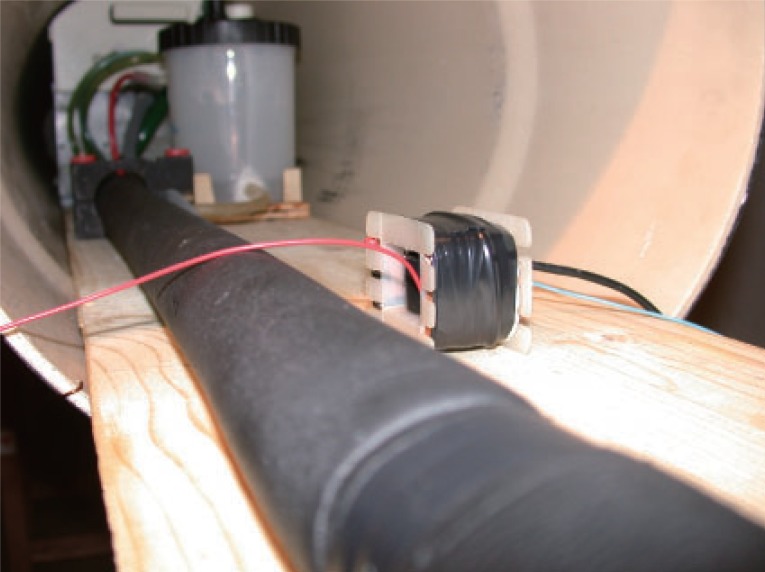
The detector and the slot for the flask.

Further the effect was searched measuring the induction in a magnetic probe due to the arising of the Zhadin effect, after the application of the combined static and magnetic field to the same solution once the proper frequency and intensity are provided as above. The electrodes were removed from the cell and substituted the cell with a flak within a condenser (Figure 5). Flask with two brass external electrodes, containing L-glutamic acid solution (33 g/l) at acid pH, was paced in the middle of a little solenoid (the probe in Figure 5) and both the flask and the probe were placed in the middle of the above big solenoid (Figure 6). The signal coming from the little solenoid was amplified and recorded.

In one set of experiments, the Zhadin effect was detected from the flask without electrodes, at the frequency of 4.99 Hz, whereas the applied static field was 49.9 μT and the alternating one was oscillating within an amplitude lower then 0.15 μT.

It is well known that Zhadin effect arises only once for each prepared solution, when the solution is kept at room temperature.

In order to investigate the relationship between temperature and arising of the effect, we stored the solution after the effect was detected at +4°C.

**Figure 7:**
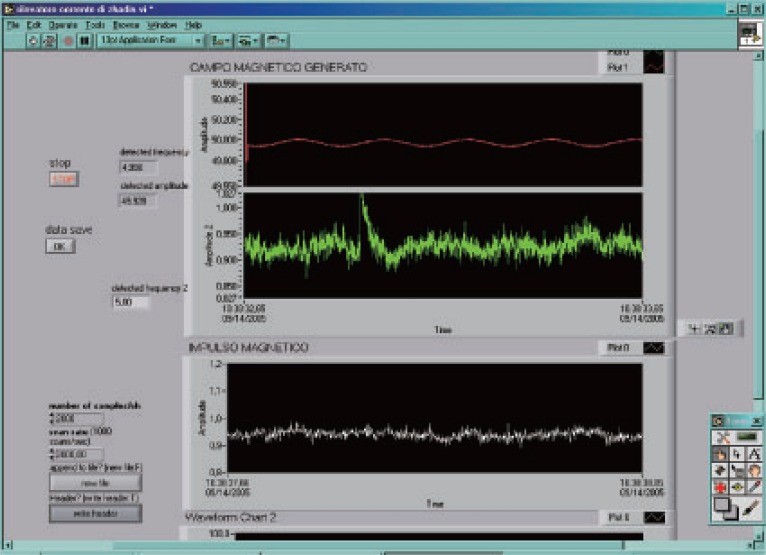
The effect detected from the flask on Sep. 14^th^.

A second set of experiments was performed using the same glutamic acid solution from the above experiments which was exposed after its temperature reached the room temperature (25°C). In this experiment the effect was detected when the applied alternating frequency was 4.99 Hz, according to the applied static magnetic field (Figure 7).

In a different set of experiment first we detected the effect one time, after refresing the solution, containing L-glutamic acid, storing it in a freezer at -80°C. After half an hour we submitted the solution to the combined static and alternating magnetic field and obtained the Zhadin effect at the alternating frequency of 2.9 Hz instead the expected frequency of 4.2 Hz (in presence of a static magnetic field of 48.9 μT).

Several replications of the above result were subsequently performed in collaboration with other researchers as previously reported (27).

Zhadin and Giuliani (20) described a mechanism concerning the arising of ions from electroneutral molecules of glutamic acid at PH 3 that are electrical dipoles (“zwitterions”). It was suggested that the transitions from the zwitterionic to the ionic form of the glutamic acid molecule increase in ion concentration more typical for pH<1. Then, at switching of the AC field matching the cyclotron frequency of glutamic acid, a weak ion current arises due to ICR, according to the schema of Del Giudice E. *et al* (15). The current is formed with the same ions that are produced during the transitions from the zwitterions to the ionic form of the glutamic acid. The ions gain the electrodes inducing polarization of the electrodes and the current ends. After the switching off of the AC field the ions near the electrodes turn ion zwitterions again due to the PH3-solution. In (20) it is suggested that the solution becomes ready to be treated again by means of AC field to produce a new ion current due to the ICR.

The model provides the explanation of the mechanism of the transitions from the zwitterionic form to the ion form of the glutamic acid before the experiment (application and tuning of the AC field inducing the arising of an ion current) and after the experiment (the back transition neighbour the electrodes from the ion from to the zwitterions form due to pH), but is not investigated if CDs are again ready to provide the energy under the form of ICER that the replication of the experiment needs. We may suppose that the CDs involved in the previous experiment are no more ready to be involved in the replication.

**Figure 8:**
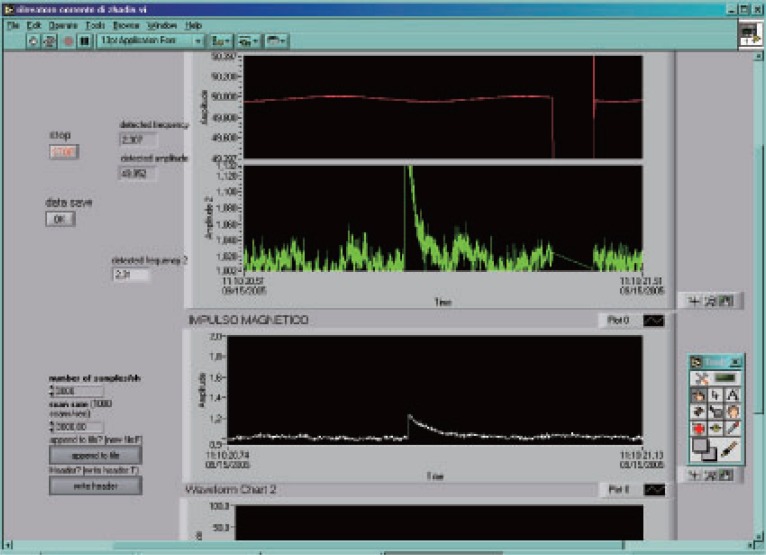
The effect after refreshing the flask at –80°C.

We were able to induce the Zhadin effect just one time: our attempts to replicate the effect after two hours, had no success at room temperature (25°C). We avoided to move the solution, leaving it in the equipment in the shielded room. After tests we decided to refresh the solution again at -80°C degrees in the same way as above. Then we put the electrolytic cell in the equipment and when its temperature reached the room temperature. We applied the AC field tuned with the cyclotron frequency of the glutamic acid and we observed the Zhadin effect (Figure 8).

## CONCLUSIONS

The temperature seems to play a peculiar role in order to detect the Zhadin effect, not only the room temperature when the experiment is performed, but the temperature when the solution is prepared or stored.

This fact provides an indirect evidence of the theory suggested by Del Giudice, Fleishman, Preparata and Talpo (15), because in the frame of the QED, we have to expect that the Coherence Dominion, whose boundary would be concerned in the experiment, growths when the temperature decreases and its boundary becomes full of ions when the temperature increases reducing the CD, in such a way the Zhadin current can arises twice in the same solution. The practical replication of the related effect twice on the same solution seems to be possible only when the solution is refreshed before the second attempt.

Another conclusion we could prospect, after we got the almost full reproducibility of the Zhadin effect: we may not consider the effect as an erratic effect and the suggested explanation in (18) would be reviewed in order to reconsider the explanation of the Zhadin effect in terms of polarization-passivation of the electrodes. This suggestion doesn’t match the evidence of the arising of the related effect when the solution is exposed in a flask not provided of internal electrodes.
